# Global explanation supervision for Graph Neural Networks

**DOI:** 10.3389/fdata.2024.1410424

**Published:** 2024-07-01

**Authors:** Negar Etemadyrad, Yuyang Gao, Sai Manoj Pudukotai Dinakarrao, Liang Zhao

**Affiliations:** ^1^Department of Electrical and Computer Engineering, George Mason University, Fairfax, VA, United States; ^2^Department of Computer Science, Emory University, Atlanta, GA, United States

**Keywords:** graph, Graph Neural Networks, global explainability, human-in-the-loop, graphical concepts

## Abstract

With the increasing popularity of Graph Neural Networks (GNNs) for predictive tasks on graph structured data, research on their explainability is becoming more critical and achieving significant progress. Although many methods are proposed to explain the predictions of GNNs, their focus is mainly on “how to generate explanations.” However, other important research questions like “whether the GNN explanations are inaccurate,” “what if the explanations are inaccurate,” and “how to adjust the model to generate more accurate explanations” have gained little attention. Our previous GNN Explanation Supervision (GNES) framework demonstrated effectiveness on improving the reasonability of the local explanation while still keep or even improve the backbone GNNs model performance. In many applications instead of per sample explanations, we need to find global explanations which are reasonable and faithful to the domain data. Simply learning to explain GNNs locally is not an optimal solution to a global understanding of the model. To improve the explainability power of the GNES framework, we propose the Global GNN Explanation Supervision (GGNES) technique which uses a basic trained GNN and a global extension of the loss function used in the GNES framework. This GNN creates local explanations which are fed to a Global Logic-based GNN Explainer, an existing technique that can learn the global Explanation in terms of a logic formula. These two frameworks are then trained iteratively to generate reasonable global explanations. Extensive experiments demonstrate the effectiveness of the proposed model on improving the global explanations while keeping the performance similar or even increase the model prediction power.

## 1 Introduction

As Deep Neural Networks (DNNs) are widely deployed in sensitive application areas, recent years have seen an explosion of research in understanding how DNNs work under the hood (e.g., explainable AI, or XAI; Adadi and Berrada, 2018; Arrieta et al., 2020) and more importantly, how to improve DNNs using human knowledge (Hong et al., 2020). In particular, Graph Neural Networks (GNNs) have been increasingly grabbed attention in several research fields, including computer vision (Fukui et al., 2019; Pope et al., 2019), natural language processing (Annervaz et al., 2018), medical domain (De Haan et al., 2009), and beyond. Such trend is attributed to the practical implication of graph data—many real-world data, such as social networks (Fan et al., 2019), chemical molecules (Scarselli et al., 2008), and financial data (Matsunaga et al., 2019), are represented as graphs.

**Figure 1:**
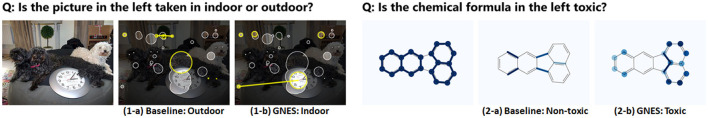
Cases for adjusting model explanation to improve Graph Neural Networks (GNNs). Scene graph **(left three)**: from the left, an input image, explanation before adjustment (1-a, inaccurate), and explanation after the adjustment (1-b, accurate). Note that the model explanation has been shifted from puppy eyes and back, rods, and an artificial tree to curtains, a clock, and a rug. Molecular formula **(right three)**: from the left, an input formula, explanation before the adjustment (2-a, inaccurate), and explanation after the adjustment (2-b, accurate). Reactivity for this molecule is mostly affected by benzene ring sub-components in the overall molecular structure. 2-b highlights the main benzene rings of the molecule more effectively than 2-a.

However, similar to other DNNs' architectures, GNNs also offer only limited transparency, imposing significant challenges in observing when GNNs make successful/unsuccessful predictions (Hong et al., 2020; Wu et al., 2021). Some real world examples for adjusting model explanation to improve Graph Neural Networks (GNNs) can be seen in Figure 1. This issue motivates a surge of recent research on GNN explanation techniques, including gradients-based methods, where the gradients are used to indicate the importance of different input features (Baldassarre and Azizpour, 2019; Pope et al., 2019); perturbation-based methods, where an additional optimization step is typically used to find the important input that influences the model output the most with input perturbations (Ying et al., 2019; Luo et al., 2020; Schlichtkrull et al., 2020); response-based methods, where the output response signal is backpropagated as an importance score layer by layer until the input space (Baldassarre and Azizpour, 2019; Pope et al., 2019; Schnake et al., 2020); surrogate-based methods, where the explanation obtained from an interpretable surrogate model that is trained to fit the original prediction is used to explain the original model (Huang et al., 2020; Vu and Thai, 2020; Zhang et al., 2020); and global explanation methods, where graph patterns are generated to maximize the predicted probability for a certain class and use such graph patterns to explain the class (Yuan et al., 2020a). Unlike local explanation models which explain the model prediction per input sample, global explanation techniques aim at providing the general insights and high-level understanding of the predictions of a deep graph model. Specifically, they investigate what input graph patterns can lead to a certain GNN behavior or maximize a certain prediction. This is essential in many real-world critical applications and can substantially increase human trust in GNNs' prediction ability. As an example, consider classifying graph molecules as either having a mutagenic effect or not (Azzolin et al., 2022). The mutagenicity of a molecule is correlated with the presence of electron-attracting elements conjugated with nitro groups (e.g., *NO*_2_). Accordingly, designing an explanation model that can provide a global understanding of the GNN classification is an urgent need. This could be achieved by designing an explainer that manages to recover all the existing well-known *NO*_2_ motifs as an indicator of mutagenicity. Additional examples include gender (male vs. female) or age (young vs. old) classification of human subjects based on structural or functional connectivity matrices, obtained through magnetic resonance imaging of the corresponding subjects. In this case, rather than a per sample explanation, we need a per class explanation in form of high-level, generic insight on differences in the input connectivity matrices of these subjects.

Despite the recent fast progress on GNN explanation techniques, the existing research body focuses on “how to generate GNN explanations” instead of “whether the GNN explanations are inaccurate,” “what if the explanations are inaccurate,” and “how to adjust the model to generate more accurate explanations.” Answering the above questions is highly beneficial to the model developers and the users of GNN explanation techniques but is also extremely difficult due to several challenges: **1) Lack of an automatic learning framework for identifying and adjusting unreasonable explanations on GNNs**. Although there are plenty of existing works on GNN explanations, they are not able to ensure the correctness of explanations, not able to identify the incorrect explanations, nor able to adjust the unreasonable explanations. The technique that can enable this has not been well-explored yet and is technically challenging due to the additional involvement of another backpropagation originated from explanation error. **2) Difficulty in aligning the node and edge explanations**. Existing GNN explanation works usually focus on either node and edge explanation, while the interplay and consistency between the explanations of nodes and edges are extremely challenging to maintain and jointly adjusted. **3) Difficulty in jointly improving model performance and explainability with limited explanation supervision**. Due to the high cost for human annotation, it can be impractical to assume the full accessibility to the human explanation label during model training. Thus, designing an effective framework that can best leverage a partially labeled dataset is on-demand yet challenging. **4) Lack of a learning framework that can employ global explanation of a GNN model to improve its performance and global explainability through global explanation supervision**. In many applications, we have access to the ground-truth explanations annotated by domain experts that can demonstrate the behavior of the data as a whole, and hence, we are motivated to employ that as the supervision signal to improve performance and global-level explainability. Designing a learning framework that utilizes this type of information is an interesting line of research which has yet remained unexplored.

To address the above challenges, beyond merely finding a solution to produce global GNN explanations, this study focuses on a global GNN explanation supervision framework for correcting the unreasonable explanations and learning how to explain GNNs from a global aspect correctly. Although the previously proposed Graph Neural Network Explanation Supervision (GNES) framework (Gao et al., 2021) has proved effective on improving the reasonability of the model explanation per local samples, while still keep or even improve the backbone GNN model performance, it still lacks the ability to guide the global model explanation generation. In many real-world decision-critical application, the ability to explain the reason for each class prediction through a single robust overview of the model is a critical requirement. To address the inefficacy of the existing GNES model in improving the global explainability through global explanation supervision, in this study, we extend the GNES model by proposing the Global GNN Explanation Supervision (GGNES), whose effectiveness is similar to the GNES model but can improve the GNN model global explanation generation (and potentially its prediction) through guiding the global explanations generated while training the model. The major contributions of this study are summarized as follows: **(1) Develop a generic framework for training GNNs while improving the reasonability and faithfulness of the global explanations generated for the model**. We propose the GGNES model built upon concept-based explainability and our previously proposed GNES model. GGNES enables learning reasonable and faithful global explanation, in terms of logic formulas, while training a GNN model. These formulas are constructed from a combination of learned graphical concepts which are derived from local explanations. **(2) Develop the formulation that can take the model-generated global node (or edge) level explanation of a GNN, and use that as additional supervision to train the GNN model**. The explanations generated by the GNN model remain differentiable to the backbone model's parameters. This makes the global explanation supervision feasible as the model parameters can be affected and tuned during training. **(3) Conduct comprehensive experiments to evaluate the effectiveness of the proposed model**. Extensive experiments on three real-world datasets demonstrate that the proposed model improved the backbone GNN model both in terms of prediction power and global explainability across different application domains. In addition, qualitative analyzes, including case studies, are provided to demonstrate the effectiveness of the proposed framework.

## 2 Related work

In this section, we first introduce our previously proposed GNES framework. Then, we note that our work draws inspiration from the research fields of graph neural network explanations that provide the model generated explanations, and explanation supervision on DNNs which enables the design of pipelines for the human-in-the-loop adjustment on the DNNs based on their explanations.

### 2.1 Our previously proposed GNES framework

In our previous study (Gao et al., 2021), we proposed a framework that learns how to jointly optimize both model prediction and model explanation by enforcing both whole graph regularization and weak supervision on model explanations. For the graph regularization, we proposed a unified explanation formulation for both node-level and edge-level explanations by enforcing the consistency between them. The node- and edge-level explanation techniques we proposed are also generic and rigorously demonstrated to cover several existing major explainers as special cases. However, in some applications, the ground truth explanations demonstrate the behavior of the data as a whole instead of each individual sample. Accordingly, we need a learning framework that utilizes this type of information through global explanation supervision and hence improves both model prediction and global model explanation.

### 2.2 Graph Neural Networks explanations

Most of the existing GNN explanation methods are instance-level methods, where the methods explain the models by identifying important input features for its prediction (Yuan et al., 2020b). The first category is gradients-based methods, where the gradients are used to indicate the importance of different input features. Existing methods are SA (Baldassarre and Azizpour, 2019), Guided BP (Baldassarre and Azizpour, 2019), CAM (Pope et al., 2019), and GradCAM (Pope et al., 2019). In Etemadyrad et al. (2022), the authors propose a novel post hoc explanation technique to find the subgraphs in input that majorly influence one or more subgraphs in the output domain by using gradient information and solving a classical community detection objective (De Domenico et al., 2015). The second category is perturbation-based methods, where an additional optimization step is typically used to find the important input that influences the model output the most with input perturbations. Existing methods are GNNExplainer (Ying et al., 2019), PGExplainer (Luo et al., 2020), and GraphMask (Schlichtkrull et al., 2020). The third category is the response-based method, where the output response signal is backpropagated as an importance score layer by layer until the input space. Existing methods in this category include LRP (Baldassarre and Azizpour, 2019), Excitation BP (Pope et al., 2019), and GNN-LRP (Schnake et al., 2020). The last category is surrogate-based methods, where the explanation obtained from an interpretable surrogate model that is trained to fit the original prediction is used to explain the original model. The surrogate methods include GraphLime (Huang et al., 2020), RelEx (Zhang et al., 2020), and PGM-Explainer (Vu and Thai, 2020). In addition to instance-level explanation methods, very recently, the global explanation of the GNN model has also been explored by XGNN (Yuan et al., 2020a). Please see Yuan et al. (2020b) for a survey of explainability in Graph Neural Networks. Even though there are plenty of existing explanation methods for GNNs, most of the methods above can not be applied to explanation supervision mechanism as the goal is to apply supervision on the generated explanation such that the backbone GNN model itself can be fine-tuned accordingly to generate better explanations as well as keep or even improve the model performance. To enable this fine-tuning process over the explanation, the explanation itself needs to be differentiable to the backbone GNN model's parameters. In other words, only the explanation that is directly calculated from the computational pipeline (such as gradients-based and response-based methods) can be used to apply this additional explanation supervision to fine-tune the backbone GNN models explanation. The perturbation-based and surrogate-based methods all require additional optimization steps to obtain the explanation and thus are unable to be end-to-end trained with the explanation supervision on the backbone GNNs.

### 2.3 Explanation supervision on DNNs

The potential of using *explanation*–methods devised for understanding which sub-parts in an instance are important for making a prediction–in improving DNNs has been studied in many domains across different applications. In fact, explanation supervision has been widely studied on image data by the computer vision community (Das et al., 2017; Linsley et al., 2018; Qiao et al., 2018; Mitsuhara et al., 2019; Zhang et al., 2019; Chen et al., 2020; Patro et al., 2020). Linsley et al. (2018) have demonstrated that the benefit of using stronger supervisory signals by teaching networks where to attend, which looks similar to the proposed approach. Moreover, Mitsuhara et al. (2019) have proposed a *post-hoc* fine-tuning strategy where an end-user is asked to manually edit the model's explanation to interactively adjust its output. Such edited explanations are then used as ground-truth explanations (from humans) to further fine-tune the model. In addition, several works in the Visual Question Answering (VQA) domain have proposed to use explanation supervision to obtain improved explanation on both the text data and the image data (Das et al., 2017; Qiao et al., 2018; Zhang et al., 2019; Patro et al., 2020). In addition to image data, the explanation supervision has also been studied on other data types, such as texts (Ross et al., 2017; Jacovi and Goldberg, 2020), attributed data (Visotsky et al., 2019), and more.

Gao et al. (2024) provide a systematic survey on Explanation-Guided Learning (EGL), a line of research that focuses on leveraging additional supervision signals or prior knowledge obtained from human explanations into machine learning models' reasoning process. According to Gao et al. (2024), EGL methods provide either global (Weinberger et al., 2020; Erion et al., 2021) or local guidance (Gao et al., 2022a,b; Shi et al., 2023)) by injecting prior knowledge or adding supervision signals to improve the model's global (or local) explanation. In Erion et al. (2021), the authors introduce attribution priors to optimize for higher-level properties of explanations, such as smoothness and sparsity. Lee et al. (2022) illustrate how to upgrade a deep model to its self explainable version that can predict and explain with logic rules learned with widely-used deep learning modules. Gupta et al. (2024) introduce Concept Distillation to create richer concepts using a pre-trained teacher model. They demonstrate how concept-sensitive training can improve model interpretability, reduce biases, and induce prior knowledge. Sha et al. (2023) propose a rational extraction technique built based on an adversarial approach that calibrates the information between a guider, a typical neural model that does the prediction, and a selector-predictor model that additionally produces a rationale for the guider' prediction. Shi et al. (2023) develop the ENGAGE framework as a local guidance EGL, built upon Explanation Guidance Data Augmentation, which leverages explanation to inform graph augmentation, and uses contrastive learning for training representations to preserve the key parts in graphs while removing uninformative artifacts.

However, to our best knowledge, explanation supervision on graph-structured data with graph neural networks through learning logic-based concepts has not been explored before, and we are the first to propose a framework to handle this open research problem.

## 3 Model

In this section, we introduce our proposed Global Explanation Supervision framework for GNNs. First, we briefly summarize the explanation regularizations (i.e., explanation consistency and sparsity) proposed by Gao et al. (2021) and how these components enhance the quality of model explanations in a global level. Then, we will introduce the proposed Global node-level, in addition to the Global edge-level explanation supervision definition and formulation.

**Formal definition of the problem:** Let G=(X,A) denotes an attributed graph with *N* nodes be defined with its node attributes $X\in {\mathbb{R}}^{N\times d_{in}}$ and its adjacency matrix *A* ∈ ℝ^*N*×*N*^ (weighted or binary), where *d*_*in*_ denotes the dimension of input feature. Let *y* be the class label for graph G. The general goal for a GNN model is to learn the mapping function *f* for each graph G to its corresponding label *y*,


F:G→y

Following Kipf and Welling (2016) and similar to Gao et al. (2021), we employ the basic definition of Graph Convolutional Networks (GCN; Kipf and Welling, 2016), for an attributed graph G=(X,A) with *y* as the class label for graph G, where a graph convolutional layer can be defined as Equation (1).


(1) $$\begin{array}{l} F^{\left(l\right)}=\sigma \left({\tilde{D}}^{-\frac{1}{2}}Ã{\tilde{D}}^{-\frac{1}{2}}F^{\left(l-1\right)}W^{\left(l\right)}\right) \end{array}$$

where *F*^(*l*)^ denotes the activations at layer *l*, and *F*^(0)^ = *X*; Ã = *A*+*I*_*N*_ is the adjacency matrix with added self connections where $I_{N}\in {\mathbb{R}}^{N\times N}$ is the identity matrix; $\tilde{D}$ is the degree matrix of Ã, where ${\tilde{D}}_{ii}=\underset{j}{\sum }{Ã}_{ij}$; The trainable weight matrix for layer *l* is denoted as *W*^(*l*)^ ∈ ℝ^*d*^^(*l*)^×*d*^(*l*+1)^; σ(·) is the element-wise non-linear activation function. Additionally, a similar design as in Pope et al. (2019) is employed to this backbone GNN model in which using several layers of graph convolutional layers followed by a global average pooling (GAP) layer over the graph nodes can address any concerns when working with variable input graph size.

### 3.1 GGNES framework

**Figure 2:**
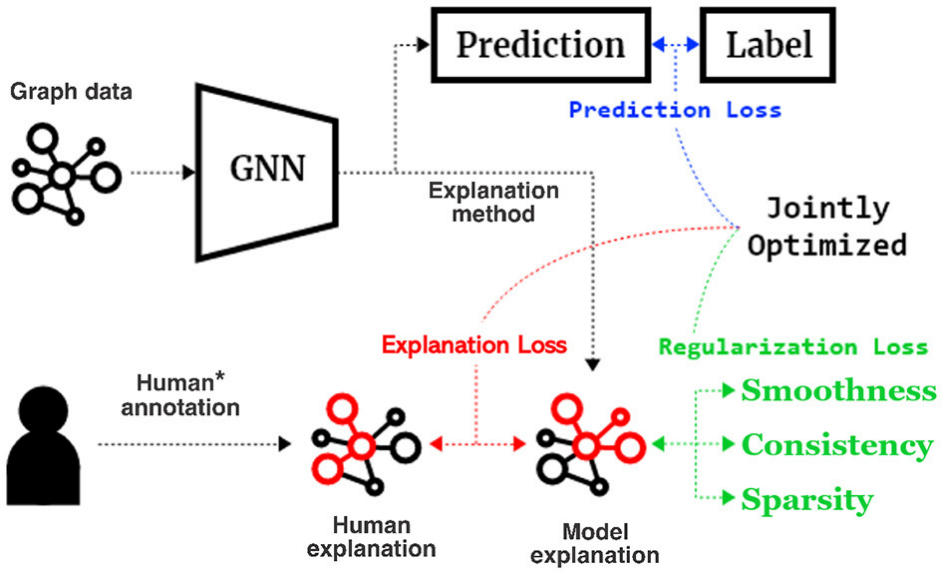
Proposed GNN Explanation Supervision (GNES) framework that jointly optimized the GNN models based on (1) a prediction loss, (2) an explanation loss on the human annotation and model explanation, and (3) a graph regularization loss to inject high-level principles of the graph-structured explanation. Notice that we only assume limited accessibility to the human annotation for only a small set of samples (10% in our experiments).

The goal here would be to design a framework that can generate global explanations which are closer to the human annotations through global explanation supervision. The prediction performance is expected to stay the same or possibly also improve. The global explanation supervision is possible via defining the learning objective of the proposed framework as a joint optimization. As shown in Equation (2) and following the framework in Figure (2), the objective function is a combination of model prediction loss (e.g., the cross-entropy loss), the global explanation loss (which is a function of the absolute or squared difference between class level human and model explanations), and global model explanation regularizations (graph regularizations that follow high-level graph-structured rules to the explanation). These three terms are computed per class and combined thereafter to form the global explanation supervision framework. Concretely, we employ the objective function as


(2) $$\begin{array}{l} \min\;L_{\text{Pred,c}}+\underset{\text{global explanation loss}}{\underset{︸}{L_{\text{Att,c}}\left(\langle M_{c},M_{c}^{\prime }\rangle ,\langle E_{c},E_{c}^{\prime }\rangle \right)}}+\underset{\text{regularization}}{\underset{︸}{\Omega_{c}\left(M_{c},E_{c}\right)}} \end{array}$$

where $M_{c}\in {\mathbb{R}}^{N\times 1}$ and $E_{c}\in {\mathbb{R}}^{N\times N}$ denote the model-generated node-level and edge-level explanations of class *c* using a given explanation method. And $M_{c}^{\prime }$, $E_{c}^{\prime }$ are the corresponding ground-truth explanations of class *c*, marked by the human annotators. The human annotations are provided globally for all samples and are unique per class, but equal for the samples of each class. These are used as additional guidance to make the explanation supervision possible. $L_{\text{Pred,c}}$ is the typical prediction loss (such as the cross-entropy loss) on the training set. The proposed explanation loss $L_{\text{Att,c}}$ measures the discrepancies between model and human explanations globally both on node level and edge level, as Equation (3)


(3) $$\begin{array}{l} L_{\text{Att}}\left(\langle M_{c},M_{c}^{\prime }\rangle ,\langle E_{c},E_{c}^{\prime }\rangle \right)=\underset{\text{global node loss}}{\underset{︸}{\alpha_{n}\text{dist}\left(M_{c},M_{c}^{\prime }\right)}}+\underset{\text{global edge loss}}{\underset{︸}{\alpha_{e}\text{dist}\left(E_{c},E_{c}^{\prime }\right)}} \end{array}$$

where α_*n*_ and α_*e*_ are the scale factors for balancing global node-level and global edge-level loss; the function dist(*x, y*) measures the mean element-wise distance between the inputs *x* and *y*, a common choose can be absolute difference or squared difference.

However, in practice, in many applications, it is not feasible to obtain the human explanations for the whole dataset. As a remedy, we only apply the global explanation loss to the classes that have the ground-truth labels for the human explanations and apply the high-level graph rules to regulate the model explanation for each class even if the human annotation is unavailable (Gao et al., 2021). Specifically, we employ the global explanation consistency, in addition to the global sparsity regularization. The former can regulate the global node and edge explanation simultaneously so that the model is more likely to generate a globally consistent and smooth explanation over nodes and edges. The global sparsity regularization is designed to regulate the model to only focus on a few important nodes and edges for the explanations. Thus, we propose Equation (4) for global graph regularizations to obtain more reasonable model explanations:


(4) $$\begin{array}{l} {\text{Ω}}_{c}\left(M_{c},E_{c}\right)=\underset{\text{explanation consistency}}{\underset{︸}{\beta {\text{Ω}}_{c}^{con}\left(M_{c},E_{c}\right)}}+\underset{\text{sparsity}}{\underset{︸}{\gamma {\text{Ω}}_{c}^{s}\left(M_{c},E_{c}\right)}} \end{array}$$

where *β* is the scaling factor for the global explanation consistency between node and edge explanations, *γ* is the scaling factor for the sparsity constraints on both node and edge explanations. These regularizations are described in more detail below:

#### 3.1.1 Global explanation consistency regularization

The global node explanation and edge explanation are not independent, but rather highly correlated with each other. One natural assumption about the global node explanation smoothness is that the adjacent nodes should share similar importance. However, this assumption can be too strong and sometimes lead to over-smoothing of the node explanation and tend to yield indistinguishable patterns for the explanation. In addition, it ignores the connection between the node and edge explanations, which can be a crucial factor for the explanation model to generate a global consistent explanation.

Here, we propose to take one step further regarding the smoothness assumption about the explanation by considering both node and edge explanations and making them more consistent with each other. Concretely, instead of treating all pairs of adjacent nodes equally important when enforcing the smoothness constraint, we propose to weight them by the corresponding edge importance such that the explanation consistency is better enforced on those nodes and edges that are deemed important. Mathematically, the global explanation consistency can be measured by Equation (5)


(5) $$\begin{array}{l} {\text{Ω}}_{c}^{con}\left(M_{c},E_{c}\right)=\frac{1}{T_{c}}\underset{k}{\sum }\frac{1}{2N^{2}}\underset{i,j}{\sum }E_{c,i,j}A_{i,j}^{k}||M_{c,i}-M_{c,j}{||}^{2} \end{array}$$

where *k* is the index of sample belonging to class *c*, $A_{i,j}^{k}$ is the adjacency matrix for sample *k*, and *T*_*c*_ is the total number of samples in class *c*. The above regularization can be interpreted as follows: given a pair of nodes *i* and *j* that is adjacent (i.e., *A*_*i, j*_ = 1), if the edge that connects the two nodes is important (i.e., *E*_*i, j*_ is high), then the nodes it connects also tend to be consistent.

#### 3.1.2 Sparsity regularization

As sparsity is a common practice for the model explanation, we apply the ℓ_1_ norm to regulate both the node-level and the edge-level explanations, as Equation (6)


(6) $$\begin{array}{l} {\text{Ω}}_{c}^{s}\left(M_{c},E_{c}\right)=\frac{1}{N}||M_{c}{||}_{1}+\frac{1}{N^{2}}||E_{c}{||}_{1} \end{array}$$

Overall, the benefits of applying the proposed regularization terms are 3-fold. First, the regularization terms do not rely on the specific human labels on the explanation, which can be very limited and hard to acquire in practice. Thus, they can be very crucial in the scenarios where the explanation labels are scarce. Second, since the explanation for the node and edge can be highly relevant, the proposed explanation consistency regularization can be critical for enforcing the model to generate more reasonable and consistent results that better align with the human explanation. Lastly, our overall framework is very flexible such that the regularization terms are not affected by changing the specification of the node and edge explanation formulation in Equations (7, 12), respectively, making the proposed framework easily applicable to give explanation and apply explanation supervision on any downstream applications with little to no overhead.

The regularization term in Equation (2) is employed to first regulate the node and edge explanation and make them consistent and smooth through considering the dependence of node and edge explanations. Additionally, to lead the model to generate more realistic explanations, the sparsity regularization is also applied which can regulate the model to only focus on a few important nodes and edges for the explanations.

### 3.2 Global node explanation formulation for global explanation supervision

In many applications, the ground-truth explanations (on synthetic data) or the domain knowledge (on real-world data) provides node-level explanation of the data in a global manner rather than per sample/instance. In this case, we need to provide a single robust overview of the model predictions. Accordingly, we aim to propose a framework that can both generate the global node explanation by capturing the behavior of the GNN model as a whole (rather than providing instance-specific explanations which could be noisy or not faithful to the model predictions) and also employ it as a supervision signal to further improve the global node-level explanations generated by the model.

To this end, we employ the gradient and the response/activation information which are also the main components for local node explanation supervision as described in Gao et al. (2021). We then aggregate this information over all instances so we can produce a model-generated global explanation that remains differentiable to the backbone GNN model's parameters. This makes the global explanation supervision feasible as the model parameters can be affected and tuned during training. Mathematically, given the output $y_{c}^{i}$ on class *c* and sample *i*, the global explanation for node *n* at layer *l* can be computed as follows:


(7) $$\begin{array}{l} M_{n,c}^{\left(l\right)}=\text{Ψ}\left(\frac{\partial y_{c}^{1}}{\partial F_{n}^{\left(l\right)}},...,\frac{\partial y_{c}^{i}}{\partial F_{n}^{\left(l\right)}},...,\frac{\partial y_{c}^{Z}}{\partial F_{n}^{\left(l\right)}},F_{n}^{\left(l\right)}\right) \end{array}$$

where $\frac{\partial y_{c}^{i}}{\partial F_{n}^{\left(l\right)}}$ represents the gradient of the features of node *n* at layer *l* given class *c* and sample *i*, *Z* is the total number of samples, and $F_{n}^{\left(l\right)}$ denotes the node activation at layer *l*. The function Ψ in Equation (7) can generate any simple to more complicated computations over the input gradients and the activation. Two simple examples are shown in Equations (8, 9), where the gradients are employed to generate simple gradient-based local explanation for each sample, which are then aggregated using the min or max function to form the final global explanation:


(8) $$\begin{array}{l} M_{n,c}^{\left(l\right)}=\min\left(||\text{ReLU}\left(\frac{\partial y_{c}^{1}}{\partial F_{n}^{\left(l\right)}}\right)||,||\text{ReLU}\left(\frac{\partial y_{c}^{2}}{\partial F_{n}^{\left(l\right)}}\right)||,...,||\text{ReLU}\left(\frac{\partial y_{c}^{Z}}{\partial F_{n}^{\left(l\right)}}\right)||\right) \end{array}$$

(9) $$\begin{array}{l} M_{n,c}^{\left(l\right)}=\max\left(||\text{ReLU}\left(\frac{\partial y_{c}^{1}}{\partial F_{n}^{\left(l\right)}}\right)||,||\text{ReLU}\left(\frac{\partial y_{c}^{2}}{\partial F_{n}^{\left(l\right)}}\right)||,...,||\text{ReLU}\left(\frac{\partial y_{c}^{Z}}{\partial F_{n}^{\left(l\right)}}\right)||\right) \end{array}$$

The other form of aggregation is to average over the local explanations to get the global-level explanation:


(10) $$\begin{array}{l} M_{n,c}^{\left(l\right)}=\frac{1}{Z}\overset{Z}{\underset{i=1}{\sum }}||\text{ReLU}\left(\frac{\partial y_{c}^{i}}{\partial F_{n}^{\left(l\right)}}\right)|| \end{array}$$

More complicated technique, described as concept-based global explainer in Azzolin et al. (2022), with some variations, can be used and formulated as below:


(11) $$\begin{array}{l} M_{n,c}^{\left(l\right)}=\text{Λ}\left(P_{1},P_{2},...,P_{m}\right) \end{array}$$

where *P*_*i*_ is the *i*−*th* learned prototype which is initialized randomly from a uniform distribution and learned through training the GLGExplainer framework described in Azzolin et al. (2022) and *m* is the total number of prototypes which is a hyperparameter and tuned separately for each dataset. Λ is also a learnable Boolean function that generates a logical combination of the learned prototypes following (Azzolin et al., 2022). In this setting, Equation (11) is a logic formula constructed using graphical concepts derived from local explanations. Concepts can be described as intermediate, high-level and semantically meaningful units of information commonly used by humans to explain their decisions. More details for GLGExplainer are given in Azzolin et al. (2022).

The training process based on Equation (11) consists of three steps. First, a basic GCN is trained by optimizing only the first term in Equation (3). Second, the local explanations generated by this trained GNN are fed as inputs to the GLGExplainer which can construct the logic formula of Equation (11). Last, the original GCN is re-trained through the full loss function in Equation (3). For the third or last step, we only employ the logic formula from step 2 and discard the prototypes generated. Instead we randomly initialize the values of prototypes from a uniform distribution. Accordingly, the GCN and GLGExplainer are trained iteratively until the value of prototypes would converge. Note that all the parameters of GLGExplainer in step three are exactly equal to those in step 2, except for the prototypes that remain learnable and are updated at each iteration.

For all the functions in Equations (8–11), the results are computed and included in the Experiments section with further discussions.

### 3.3 Global edge explanation formulation for global explanation supervision

While several works have studied global node-level explanation topic, little to no work has explored the global edge-level explanation and its applications. However, in many scenarios, the latter can be more crucial and meaningful than the former as the domain knowledge or human annotations describe the relationship between nodes rather than the nodes in particular.

Similar to the global node explanation supervision, we need to propose a unified edge-level explanation formulation which generates explanations that are differentiable to the backbone model's parameters. Taking the gradient of each edge in the input adjacency matrix, as well as the response/activation of the pairs of nodes that are associated with that edge, and using the chain rule, we can define suitable model generated explanations for each instance. Concretely, given the output $y_{c}^{i}$ on class *c* and sample *i*, the global edge explanation between node *n* and node *m* at layer *l* can be computed as the aggregation of all edge explanations for single instances. More precisely, this is a function of the edge gradients for all samples, in addition to node activations:


(12) $$\begin{array}{l} E_{n,m,c}^{\left(l\right)}=\Phi (\frac{\partial y_{c}^{1}}{\partial F^{\left(l\right)}}\cdot \frac{\partial F^{\left(l\right)}}{\partial A_{n,m}^{1}},...,\frac{\partial y_{c}^{i}}{\partial F^{\left(l\right)}}\cdot \frac{\partial F^{\left(l\right)}}{\partial A_{n,m}^{i}},\\ \;..,\frac{\partial y_{c}^{N}}{\partial F^{\left(l\right)}}\cdot \frac{\partial F^{\left(l\right)}}{\partial A_{n,m}^{N}},F_{n}^{\left(l\right)},F_{m}^{\left(l\right)}) \end{array}$$

where $\frac{\partial y_{c}^{i}}{\partial F^{\left(l\right)}}\cdot \frac{\partial F^{\left(l\right)}}{\partial A_{n,m}^{i}}$ represents the gradient of the edge that connects nodes *n* and *m* at layer *l* given class *c* and sample *i*; $F_{n}^{\left(l\right)}$ and $F_{m}^{\left(l\right)}$ denote the activation of node *n* and node *m* at layer *l*, respectively, and *N* is the total number of instances. Similar to previous formulation in Equation (7), Φ can combine the local explanations of all samples, providing a global explanation for the overall behavior of the GNN. A simple example is the min or max value among all the gradient-based edge-level local explanations, which can be formulated as Equation (13)


(13) $$\begin{array}{l} E_{n,m,c}^{\left(l\right)}=\min(||\text{ReLU}(\frac{\partial y_{c}^{1}}{\partial F^{\left(l\right)}}\cdot \frac{\partial F^{\left(l\right)}}{\partial A_{n,m}^{1}})||,...,\\ \;||\text{ReLU}(\frac{\partial y_{c}^{i}}{\partial F^{\left(l\right)}}\cdot \frac{\partial F^{\left(l\right)}}{\partial A_{n,m}^{i}})||,...,\\ \;||\text{ReLU}(\frac{\partial y_{c}^{Z}}{\partial F^{\left(l\right)}}\cdot \frac{\partial F^{\left(l\right)}}{\partial A_{n,m}^{Z}})||) \end{array}$$

where *Z* is the total number of samples, and a similar formulation can be used to find the max value of the local explanations. Averaging over the local explanations can also be another aggregator to generate the global .explanation and can be shown by Equation (14)


(14) $$\begin{array}{l} E_{n,m,c}^{\left(l\right)}=\frac{1}{Z}\overset{Z}{\underset{i=1}{\sum }}||\text{ReLU}\left(\frac{\partial y_{c}^{i}}{\partial F^{\left(l\right)}}\cdot \frac{\partial F^{\left(l\right)}}{\partial A_{n,m}^{i}}\right)|| \end{array}$$

Similar to Equation (11), the global edge explanation can also be represented as a learnable logic combination of concepts. As long as the GNN model can generate local explanations that are a subgraph of the input data, these can be fed into the GLGExplainer in Azzolin et al. (2022) which can learn the formula, and parameters in Equation (11) and generate the global explanation per class.

These various functions for Φ are investigated in detail in the Experiment section.

## 4 Experiments

We test our Global GNN Explanation Supervision framework on the datasets extracted from two publicly available sources including HCP (Human Connectome Project) and the ABIDE (Autism Brain Imaging Data Exchange) database. These datasets, in addition to the implementation details, evaluation metrics, and comparison methods are described in turn below.

### 4.1 Datasets

#### 4.1.1 Magnetic resonance imaging data

The (structural, diffusion, and functional) MRI data were extracted from the Human Connectome Project website (https://db.humanconnectome.org/), specifically, the 1,200 Subjects Release, February 2017 (Van Essen et al., 2013), which provided (MRI) data from 1,200 young adult (ages 22–35) subjects. Here, two tasks are defined as binary classification of a given subject as Female vs. Male, in addition to Young (22–29) vs. Old (29–35). The age and gender labels were provided as additional meta features. For the ground-truth explanations of each class, we refer to Gong et al. (2009), which has investigated age and sex effects on the anatomical connectivity patterns of 95 normal subjects ranging in age from 19 to 85 years. Accordingly, cortical regions which show significant effect for young, old, male, or female subjects were separately identified for each group for Automated Anatomical Labeling (AAL) atlas (Tzourio-Mazoyer et al., 2002). To use these as annotations for HCP dataset, these regions were then mapped to Desikan-Killiany (DK) atlas (Desikan et al., 2006), by finding the closest node (Euclidean distance) in DK to each identified node in AAL atlas. The resulting DK nodes are provided in Supplementary material for each class under study.

The raw MRI data were then preprocessed using the HCP pipeline (WU-Minn, 2017). For the diffusion MRI, this was followed by the BEDPOSTX (Bayesian Estimation of Diffusion Parameters Obtained using Sampling Techniques, modeling crossing X fibers) algorithm in the FMRIB Software Library (Jenkinson et al., 2012, FSL), which models white matter fiber orientations and crossing fibers for probabilistic tractography. The resting state blood-oxygen-level-dependent functional MRI (r-fMRI) time series data were acquired from participants, in four runs of ~15 min for each participant, including two runs on two different days (Day 1 and Day 2). These measurements were collected with the subject supine and still, with eyes open, to track physiological changes in the brain (i.e., changes in blood flow and oxygen levels) that occur in resting state, when an explicit task is not being performed (Biswal, 2012; Buckner et al., 2013).

**Extracting SC and FC:** To construct the SC matrix for each subject, we ran Probtrackx in FSL with 68 regions of interest (ROIs) obtained from the the DK atlas. For the remaining parameter setting in Probtractx, we followed the recommendations of the tutorial (in St.Louis, 2020) provided by HCP. Finally, the resulting SC matrices were normalized by dividing the respective row sum from each non-zero value.

Three steps were followed to extract the functional connectivity from the r-fMRI time series data, for each day: 1. Concatenate the time series for the two runs together; 2. For each of the 68 ROIs defined by the Desikan-Killiany atlas, average all the time series to create a single ROI time series; and 3. obtain the functional connectivities by either (a) Computing the pairwise ROI time series' Pearson correlations using FSLNets (of Heidelberg Department of Neuroradiology, 2014) with the full correlation option, thus generating **Dataset 1**; or following similar three steps as mentioned for Dataset 1, except that we Concatenate the time series for the two runs performed in day 2 together, thus generating **Dataset 2**. In this study, we followed with the experiments only using **Dataset 1** due to the high amount of computation and resources required for each Dataset.

#### 4.1.2 ABIDE dataset

We analyzed r-fMRI in the Autism Brain Imaging Data Exchange (ABIDE; Di Martino et al., 2014). It compiles a dataset of 1,112 r-fMRI participants by gathering data from 16 international imaging sites that have aggregated and are openly sharing neuroimaging data from 539 individuals suffering from ASD and 573 typical controls (TCs). The task is to classify a subject as either belonging to ASD or the control group, based on their r-fMRI data. Since there was no prior coordination between sites, the scan and diagnostic/assessment protocols vary across sites. Accordingly, we rely on a publicly available preprocessed version of this dataset provided by the Preprocessed Connectome Project (PCP) initiative. PCP preprocessed the data using four different pipelines, all of which implemented fairly similar steps, but varied in the algorithms used for each step and the parameters. We specifically used the data processed with the Configurable Pipeline for the Analysis of Connectomes, C-PAC (Craddock et al., 2013), which provides further minimally preprocessed data through the python package, cpac. C-PAC comes pre-packaged with a default pipeline, as well as a growing library of pre-configured pipelines. These pipelines could be edited or built from scratch, using the provided pipeline builder. For our experiments, we used the default processing pipeline. For more details, please see Craddock et al. (2013) on how we extracted time series for the Harvard-Oxford atlas. We finally used the same steps as HCP dataset, to compute the functional connectivity matrices. Additionally, we used the biomarkers extracted by Kunda et al. (2020), as the ground-truth labels for explanation supervision. These include the top five most contributing FC edges for ASD and TC classification, respectively (10 overall connections), built using the Harvard-Oxford (HO) brain atlas (Jenkinson et al., 2012) as a point of reference.

### 4.2 Implementation details

Following the previous work on the explanation supervision for GNNs, we used a 3 layer GCN as our backbone GNN model. The hidden dimension size for the three graph convolutional layers is tuned separately for each dataset/task. We used 2 for Gender prediction and 3 for age prediction tasks. For the ASD classification task, we found 3 to best classify the dataset. These hidden layers are followed by a global average pooling (GAP) layer, and a softmax classifier. Models were trained for 200, 300, and 260 epochs using the ADAM optimizer (Kingma and Ba, 2014), respectively, with a learning rate of 0.001 in all three cases. For the remaining details of implementation and parameters, we followed all the settings in Gao et al. (2021), unless otherwise specified.

For the GLGExplainer, we prepared the input using the simple gradient-based local explainer in the backbone GNN. The number of prototypes was set to 4 and 2 for the HCP dataset and the ABIDE data, respectively. This explainer was trained using all the remaining settings and parameters including the optimizer, learning rate, batch size, focusing parameter, and auxiliary loss coefficients, in addition to the E-LEN, from the original proposed model (Azzolin et al., 2022).

#### 4.2.1 Evaluation metrics

We evaluate the effectiveness of the proposed GGNES model in terms of prediction performance as well as in terms of global explainability. Specifically, for model performance assessment, we use accuracy (ACC) and Area Under the Curve (AUC) scores to measure the prediction power of the GNNs on the prediction tasks for all the datasets. In addition, we leverage the human/domain-labeled explanation on the test set to quantitatively assess the goodness of the model explanation. Specifically, for both node-level and edge-level global explanations, we treat the human explanation as the gold standard and compute the distance between human and global model explanation via Mean Square Error (MSE) and Mean Absolute Error (MAE). Additionally, we evaluate our model on: (i) FIDELITY, which represents the accuracy of the E-LEN in matching the predictions of the GNN model to explain; (ii) ACCURACY, which represents the accuracy of the formulas in matching the ground-truth labels of the graphs; (iii) CONCEPT PURITY, which is computed for every cluster independently and measures how good the embedding is at clustering the local explanations (Azzolin et al., 2022), and is computed through Equation (15)


(15) $$\begin{array}{l} \text{ConceptPurity}\left(C_{i}\right)=\frac{\text{count\_most\_frequent\_label}\left(C_{i}\right)}{\left|C_{i}\right|} \end{array}$$

where *C*_*i*_ corresponds to the cluster having *p*_*i*_ as the learned prototype, and *count*_*most*_*frequent*_*label*(*C*_*i*_) returns the number of local explanations annotated with the most present label in cluster *C*_*i*_. The Concept Purity results are reported by computing the mean and the standard deviation across all clusters. For a more detailed description of these metrics, see Azzolin et al. (2022).

#### 4.2.2 Comparison methods

Since there is no existing work on global explanation supervision on GNNs, we demonstrate the effectiveness of our model by comparing the evaluation metrics in the following scenarios:

- No explanation supervision technique is used.
- min, max, or Average functions are used to generate global explanation and perform explanation supervision.
- Concept-based global explanation is constructed and further used for supervision.

### 4.3 Experimental results

**Figure 3:**
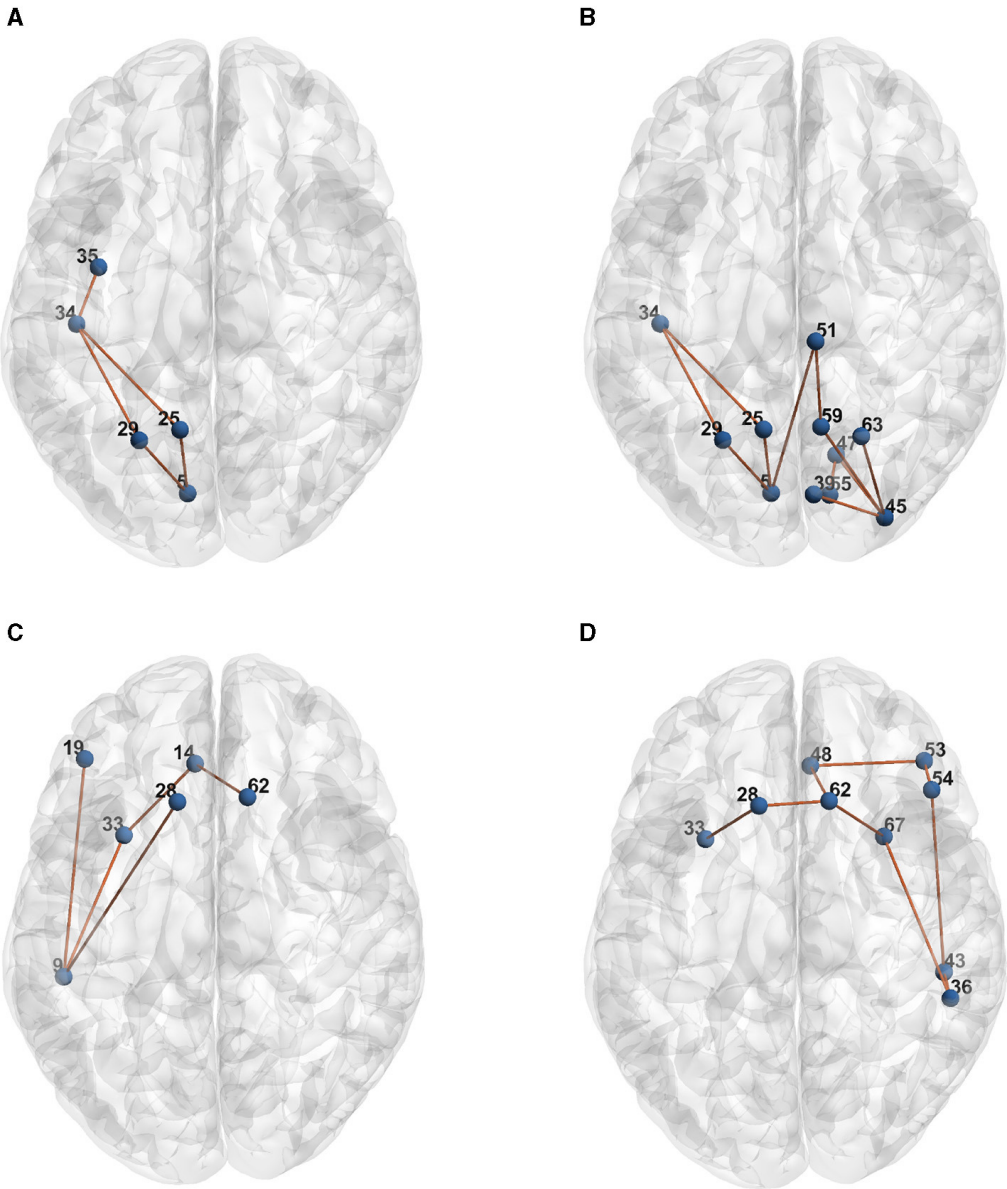
Representative element of each learned concept for HCP Structural dataset. **(A)** *P*_0_. **(B)** *P*_1_. **(C)** *P*_2_. **(D)** *P*_3_.

**Figure 4:**
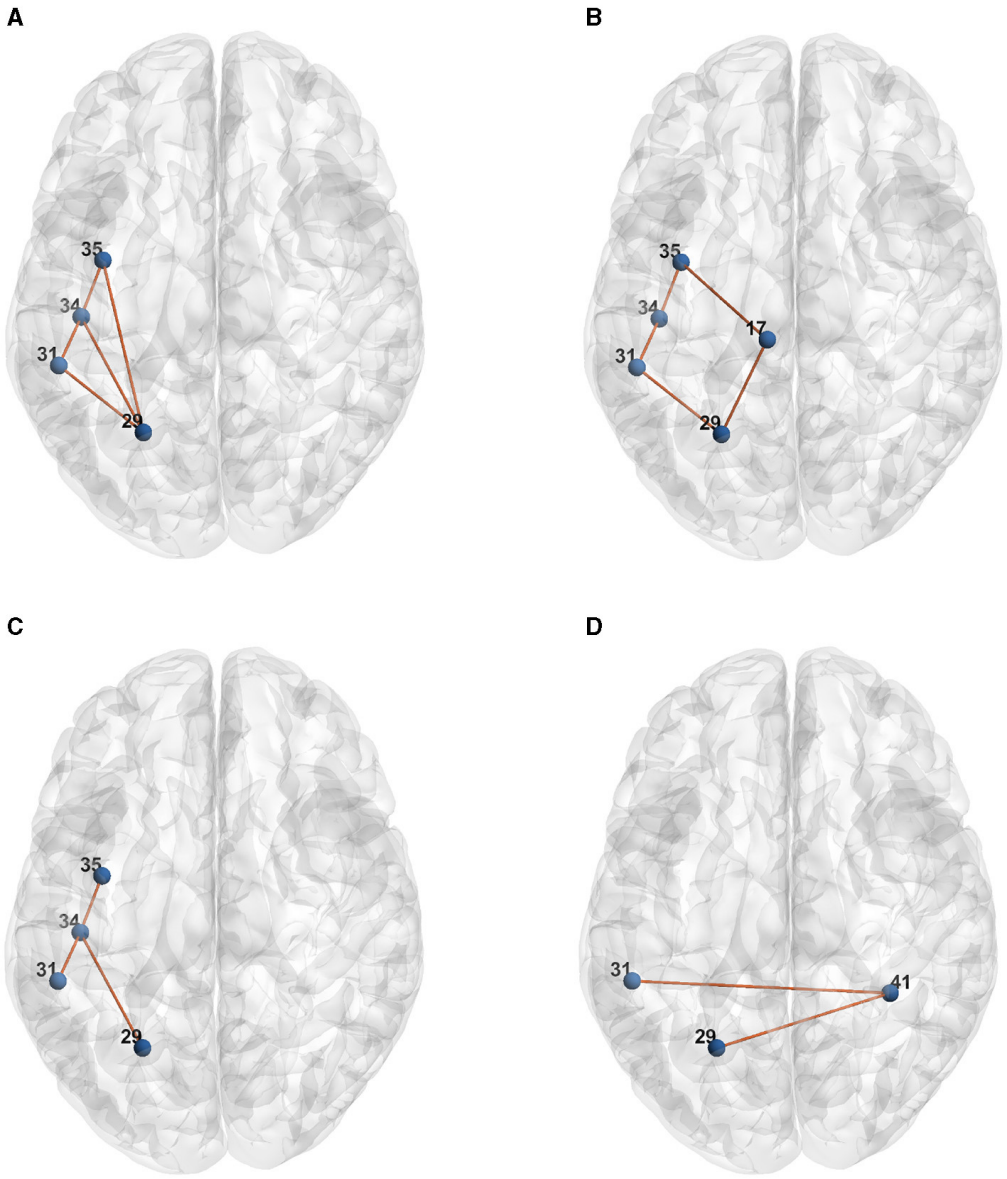
Representative element of each learned concept for HCP Functional dataset. **(A)** *P*_0_. **(B)** *P*_1_. **(C)** *P*_2_. **(D)** *P*_3_.

**Figure 5:**
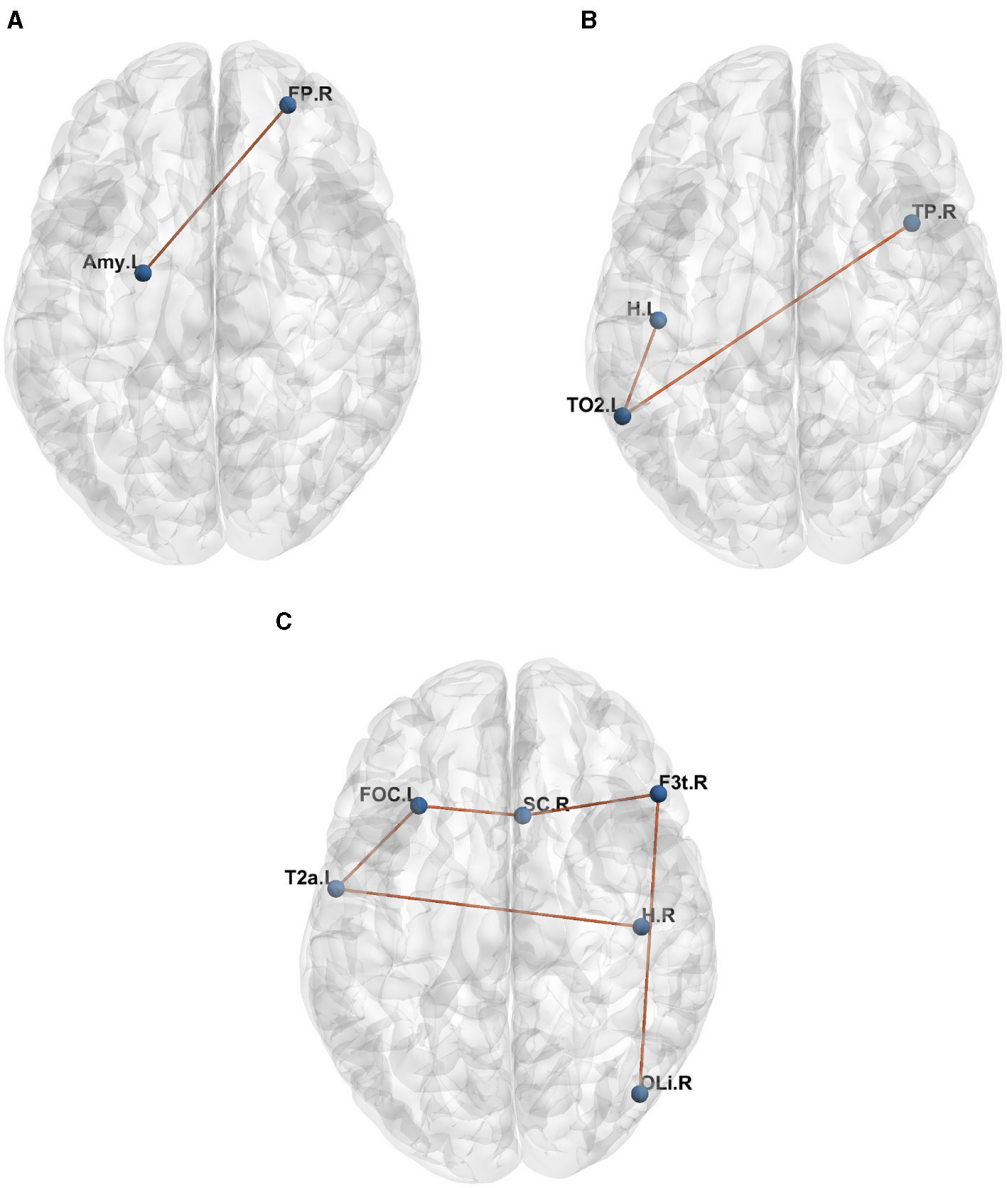
Representative element of each learned concept for ABIDE dataset. **(A)** *P*_0_. **(B)** *P*_1_. **(C)** *P*_2_.

Table 1 presents the raw formulas extracted by the Entropy Layer. Those formulas can be further described in a more human-understandable format after finding the representative elements of each cluster as shown in Figures 3–5, which correspond to HCP structural, HCP functional, and ABIDE datasets, respectively. Each of these Figures contains a number of sub-Figures that show the learned prototypes described in Section 3.2. Specifically, for each prototype *p*_*j*_, the local explanation Ḡ such that $Ḡ={\mathrm{argmax}}_{{Ḡ}^{\prime }\in D}d(p_{j},h\left({Ḡ}^{\prime }\right)$ is reported. Here, *D* is a list of local explanations obtained after the binarization step in GLGExplainer. For details on this step, in addition to the definition for distance function *d*(), please see Azzolin et al. (2022). The nodes in Figures 3, 4 refer to DK atlas and are labeled with numbers for better readability, while the nodes in Figure 5 correspond to HO atlas. See Supplementary material for the label names corresponding to the labels we used in Figures 3, 4. For a list of HO atlas labels used in Figure 5, see Atlas (2023).

**Table 1:** Raw formulas as extracted by the Entropy Layer.

**Dataset**	**Task**	**Raw formulas**
HCP structural	Gender prediction	Female ⇔*P*_0_∨*P*_1_∨*P*_2_
		Male ⇔*P*_3_
HCP functional	Age prediction	Old ⇔ *P*_0_∨(*P*_1_∧*P*_2_)
		Young ⇔ *P*_2_∨*P*_3_
ABIDE	ASD classification	Typical ⇔ *P*_0_∧*P*_1_
		Control ⇔ *P*_2_

#### 4.3.1 Performance

Table 2 shows the model performance and model-generated explanation quality for the three described datasets. The results are obtained from 5 individual runs for every setting. The best results for each dataset are highlighted with boldface font, and the second bests are underlined. For the HCP datasets, for both Age and Gender prediction tasks, the human annotations contain only node-level explanations, but for the ABIDE dataset we have both ground-truth (domain-labeled) node-level and edge-level explanations available for all samples. In general, our proposed Global Explanation Supervision model variations outperformed the non-explanations supervision GNN model in terms of both prediction power as well as explainability on all three datasets. More specifically, the performance results for different variations suggested that global explanation supervison can have positive effects in all scenarios on both prediction power, in addition to the explanation correctness. The most complicated model (i.e., the concept-based supervision model) achieved the best performance, out-performing baseline GNN by 1–6% and 1–3% on AUC and ACC scores, respectively. In addition, in terms of explainability, there is significant improvement in both node and edge-level explanations, when comparing the backbone GNN and the concept-based supervision models. In particular, we observed between 19–57% increase in node MSE and 14–48% in node MAE, and more than 33% improvement for edge MSE and MAE explanations.

**Table 2:** Performance and model-generated explanation evaluation among the proposed models and the baseline on two HCP, in addition to one ABIDE graph classification tasks.

**Dataset**	**Global_exp _method**	**ACC**	**AUC**	**Node MSE**	**Node MAE**	**Edge MSE**	**Edge MAE**
HCP functional	None	0.736	0.843	0.392	0.436	–	–
	Avg	0.741	0.854	**0.311**	0.394	–	–
	max	0.736	0.843	0.324	0.418	–	–
	min	0.738	0.845	0.321	0.414	–	–
	concept_based	**0.759**	**0.899**	**0.311**	**0.372**	–	–
HCP structural	None	0.829	0.961	0.238	0.436	–	–
	Avg	0.833	0.965	0.224	0.322	–	–
	max	0.830	**0.971**	0.220	0.397	–	–
	min	0.833	**0.971**	0.217	0.323	–	–
	concept_based	**0.838**	**0.971**	**0.101**	**0.223**	–	–
ABIDE	None	0.730	0.868	0.237	0.437	0.065	0.033
	Avg	0.735	0.870	0.218	0.416	0.051	0.031
	max	0.732	0.871	0.215	0.406	0.055	0.025
	min	0.730	0.868	0.222	0.413	0.061	**0.021**
	concept_based	**0.744**	**0.885**	**0.191**	**0.331**	**0.043**	0.024

The results are obtained from five individual runs for every setting. The best results for each task are highlighted with boldface font, and the second bests are underlined.

These results demonstrate the general effectiveness of the proposed framework both on largely correcting the model-generated global explanation, in addition to improving the model performance and prediction power. In addition, among different variations used for global explanation generation, we observe constant superiority of the more sophisticated concept-based technique compared to the others, while no clear excellence of Avg, max, or min methods when comparing one to the other was remarkable.

To further evaluate the extracted global explanation formulas presented in Table 1, we computed Fidelity, Accuracy, and Concept Purity over the test set. The results are reported in Table 3 for the three datasets. As it can be seen, on average, the clusters are quite homogeneous, which means the model has learned a good mapping from the local explanations to the concepts space. Also the concept purity is at its lowest for HCP structural dataset while has the highest value for the same set. The accuracy results demonstrate that the formula in Table 1 can correctly match the behavior of the model in most samples. Additionally, it is important to note that by looking at the fidelity results, it is clear that the explainer is generating an explanation for the ground-truth labeling of the dataset, while capturing the underlying predictive behavior of the GNN it is supposed to explain.

**Table 3:** Fidelity, accuracy, and concept purity computed over test sets for all datasets.

**Dataset**	**Fidelity**	**Accuracy**	**Concept purity**
HCP structural	0.91	0.89	0.82
HCP functional	0.81	0.83	0.85
ABIDE	0.78	0.79	0.85

#### 4.3.2 Effect of choice of local explainer and backbone GNN model

To evaluate the proposed model more comprehensively, we repeated experiments for the model performance and model-generated explanation quality for all datasets for two additional local explanation techniques, Guided BP and GradCAM, and one other backbone GNN model, DGCNN (Zhang et al., 2018). The results are shown in Tables 4, 5. As these results show, we continue to see superiority of our proposed Global Explanation Supervision model variations compared to non-explanation supervised scenarios. The concept-based supervision model again achieved the best performance, out-performing baseline GNN, for the two backbone GNNs and all three local explanation techniques. Additionally, we observe significant improvement in both node, and edge-level MSE and MAE, when using the concept-based supervision models. These improvements, both in explanation quality and model performance, for the concept-based technique largely exceed other simple aggregation methods (e.g., Averaging) in almost all settings as well.

**Table 4:** Performance and model-generated explanation evaluation for two additional local explainers with GCN as the backbone model.

**Dataset**	**Local_exp _method**	**Global_exp _method**	**ACC**	**AUC**	**Node MSE**	**Node MAE**	**Edge MSE**	**Edge MAE**
HCP functional	Guided BP	None	0.736	0.843	0.392	0.436	–	–
		Avg	0.736	0.845	0.341	0.381	–	–
		max	0.736	0.843	0.355	0.410	–	–
		min	0.739	0.845	0.321	0.382	–	–
		concept_based	**0.752**	**0.870**	**0.311**	**0.377**	–	–
		Grad-CAM	None	0.736	0.843	0.392	0.436	–	–
	Avg		0.738	0.854	**0.311**	0.384	–	–
	max		0.737	0.845	0.338	0.422	–	–
	min		0.736	0.845	0.348	0.417	–	–
	concept_based		**0.749**	**0.893**	**0.311**	**0.362**	–	–
HCP structural	Guided BP	None	0.829	0.961	0.238	0.436	–	–
		Avg	0.831	0.965	0.211	0.289	–	–
		max	0.833	**0.970**	0.224	0.318	–	–
		min	0.835	0.966	0.221	0.314	–	–
		concept_based	**0.840**	**0.970**	**0.118**	**0.233**	–	–
		Grad-CAM	None	0.829	0.961	0.238	0.436	–	–
	Avg		0.835	0.966	0.188	0.239	–	–
	max		0.833	**0.968**	0.124	0.228	–	–
	min		0.829	0.965	0.201	0.314	–	–
	concept_based		**0.838**	**0.968**	**0.111**	**0.225**	–	–
ABIDE	Guided BP	None	0.730	0.868	0.237	0.437	0.065	0.033
		Avg	0.735	0.873	0.230	0.416	0.055	0.030
		max	0.732	0.874	0.227	0.416	0.053	0.025
		min	0.730	0.869	0.222	0.403	0.061	**0.027**
		concept_based	**0.744**	**0.883**	**0.200**	**0.355**	**0.045**	**0.027**
		Grad-CAM	None	0.730	0.868	0.237	0.437	0.065	0.033
	Avg		0.735	0.878	0.218	0.403	**0.045**	**0.023**
	max		0.730	0.869	0.218	0.392	0.055	0.026
	min		0.730	0.868	0.212	0.412	0.061	0.026
	concept_based		**0.741**	**0.885**	**0.195**	**0.337**	**0.045**	0.025

The results are obtained from five individual runs for every setting. The best results for each task are highlighted with boldface font, and the second bests are underlined.

**Table 5:** Performance and model-generated explanation evaluation for all three local explainers with DGCNN as the backbone model.

**Dataset**	**Local_exp _method**	**Global_exp _method**	**ACC**	**AUC**	**Node MSE**	**Node MAE**	**Edge MSE**	**Edge MAE**
HCP functional	Gradient based	None	0.708	0.791	0.394	0.438	–	–
		Avg	0.715	0.809	0.343	0.398	–	–
		max	0.708	0.792	0.344	0.402	–	–
		min	0.712	0.812	0.332	0.422	–	–
		concept_based	**0.718**	**0.855**	**0.301**	**0.328**	–	–
		Guided BP	None	0.708	0.791	0.394	0.438	–	–
	Avg		0.712	0.832	0.342	0.400	–	–
	max		0.712	0.838	0.339	0.400	–	–
	min		0.708	0.805	0.337	0.391	–	–
	concept_based		**0.715**	**0.861**	**0.319**	**0.370**	–	–
		Grad-CAM	None	0.708	0.791	0.394	0.438	–	–
	Avg		0.721	0.844	0.330	**0.375**	–	–
	max		0.708	0.843	**0.308**	0.390	–	–
	min		0.708	0.835	0.320	0.385	–	–
	concept_based		**0.725**	**0.857**	0.315	**0.375**	–	–
HCP structural	Gradient based	None	0.803	0.941	0.279	0.446	–	–
		Avg	0.812	0.954	0.189	**0.297**	–	–
		max	0.803	0.943	0.224	0.318	–	–
		min	0.808	0.945	0.185	0.314	–	–
		concept_based	**0.812**	**0.958**	**0.145**	0.298	–	–
		Guided BP	None	0.803	0.941	0.279	0.446	–	–
	Avg		0.808	0.953	0.231	0.294	–	–
	max		0.808	0.954	0.198	0.318	–	–
	min		0.803	0.945	0.221	0.314	–	–
	concept_based		**0.814**	**0.961**	**0.161**	**0.286**	–	–
		Grad-CAM	None	0.803	0.941	0.279	0.446	–	–
	Avg		0.811	0.962	0.201	0.304	–	–
	max		0.806	0.953	0.228	0.388	–	–
	min		0.803	0.952	0.220	0.401	–	–
	concept_based		**0.815**	**0.963**	**0.183**	**0.272**	–	–
ABIDE	Gradient based	None	0.730	0.860	0.292	0.446	0.065	0.033
		Avg	0.741	0.868	0.221	0.394	0.045	**0.026**
		max	0.741	0.867	0.254	0.418	0.045	**0.026**
		min	0.732	0.865	0.271	0.392	0.054	0.029
		concept_based	**0.744**	**0.874**	**0.211**	**0.372**	**0.043**	**0.026**
		Guided BP	None	0.730	0.860	0.292	0.446	0.065	0.033
	Avg		0.737	0.873	0.199	0.362	**0.041**	0.026
	max		0.732	0.865	0.198	0.400	0.051	**0.023**
	min		0.732	0.867	0.198	0.403	0.053	0.027
	concept_based		**0.742**	**0.880**	**0.197**	**0.343**	0.048	0.026
		Grad-CAM	None	0.730	0.860	0.292	0.446	0.065	0.033
	Avg		0.738	0.873	0.221	0.401	0.051	0.027
	max		0.734	0.872	0.221	0.398	0.056	0.027
	min		0.735	0.870	0.219	0.415	0.060	0.028
	concept_based		**0.744**	**0.883**	**0.193**	**0.335**	**0.043**	**0.025**

The results are obtained from five individual runs for every setting. The best results for each task are highlighted with boldface font, and the second bests are underlined.

#### 4.3.3 Qualitative analysis: case studies

**Figure 6:**
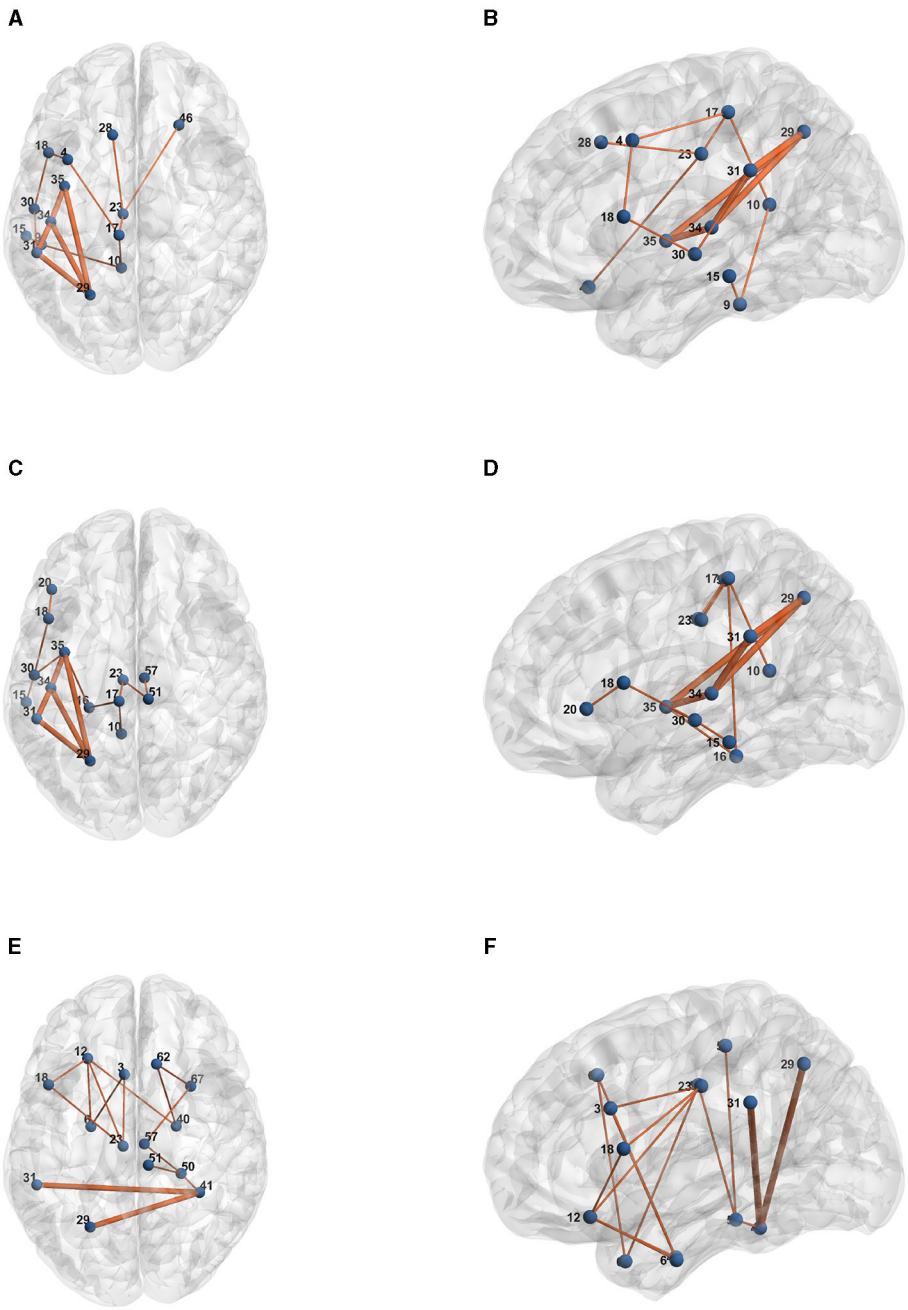
Examples of input graphs with their explanations in bold as extracted by Gradient-based edge explanation technique, for HCP structural connectivity dataset. **(A)** Subject 286-Female-Axial view. **(B)** Subject 286-Female-Sagittal view. **(C)** Subject 543-Female-Axial view. **(D)** Subject 543-Female-Sagittal view. **(E)** Subject 56-Male-Axial view. **(F)** Subject 56-Male-Sagittal view.

Here, we provide some case studies of the input data and the model explanation derived from gradient based explanation technique and binarized following (Azzolin et al., 2022). We report some random examples for each dataset, with their extracted explanation in bold, as illustrated in Figure 6.

## 5 Conclusion

In this study, we address an existing challenge for explainability in GNNs, by proposing the Global GNN Explanation Supervision (GGNES) technique which uses a basic trained GNN and a global extension of the loss function used in the GNES framework. This GNN creates local explanations which are fed to a Global Logic-based GNN Explainer, an existing technique that can learn the global Explanation in terms of a logic formula. These two frameworks are then trained iteratively to generate reasonable global explanations. Extensive experiments demonstrate the effectiveness of the proposed model on improving the global explanations while keeping the performance similar or even increase the model prediction power.

## Data availability statement

The original contributions presented in the study are included in the article/Supplementary material, further inquiries can be directed to the corresponding author.

## Ethics statement

The studies involving humans were approved by the Autism Brain Imaging Data Exchange, the Human Connectome Project. The studies were conducted in accordance with the local legislation and institutional requirements. The participants provided their written informed consent to participate in this study.

## Author contributions

NE: Conceptualization, Data curation, Formal analysis, Methodology, Visualization, Writing—original draft, Writing—review & editing. YG: Methodology, Writing—original draft, Writing—review & editing. SM: Resources, Writing—original draft, Writing—review & editing. LZ: Conceptualization, Formal analysis, Methodology, Writing—original draft, Writing—review & editing.
