# Supplementary material for: Global explanation supervision for Graph Neural Networks
Source: Front Big Data. 2024 Jul 1;7:1410424. doi: 10.3389/fdata.2024.1410424 (PMC11246961; doi:10.3389/fdata.2024.1410424)
Supplement: Supplementary file 1 [file Data_Sheet_1.PDF]

Supplementary Material

1 AAL NODES WITH SIGNIFICANT IMPACT ON AGE AND GENDER BINARY CLASSIFICATION

Here, we report the ground truth brain regions in AAL atlas (Tzourio-Mazoyer et al., 2002), which significantly contribute in determining age and gender, according to (Gong et al., 2009). L and R represent left and right, respectively. For the abbreviations of cortical regions, see (Tzourio-Mazoyer et al., 2002).

Class	Region
Young	ROL.L
	HES.L
	STG.L
	PCUN.L
	SPG.L
	SPG.R
	PCL.R
	PCUN.R
	CUN.R
	CUN.L
	SOG.R
	CAL.R
	SOG.L
	LING.R
	IOG.R

Table S1. AAL regions with significant age effect.

Class	Region
Old	ORBmid.L
	SFGdor.L
	ORBsup.R
	ORBmid.R
	SMA.L
	ORBsup.L
	IFGtriang.R
	SFGdor.R
	ITG.L
	ITG.R
	MTG.R
	TPOsup.R
	TPOmid.R
	TPOmid.L

Table S2. AAL regions with significant age effect.

Class	Region
Female	HES.L
	STG.L
	SPG.L
	IPL.L
	FFG.R
	INS.L

Table S3. AAL regions with significant gender effect.

Class	Region
Male	ROL.R
	IFGtriang.R

Table S4. AAL regions with significant gender effect.

2 DESIKAN-KILLIANY ATLAS LABELS

The region names corresponding to labels in Figures 3, 4, and 6 in main content, are shown in Figure S1, retrieved from (Jao et al., 2021).

Frontal	ROI	Abbreviation	Temporal	ROI	Abbreviation
1, 2	Caudal middle frontal	CMF	33, 34	Bankssts	B
3, 4	Frontal pole	FPol	35, 36	Entorhinal	En
5, 6	Lateral orbitofrontal	LOrF	37, 38	Inferior temporal	IT
7, 8	Medial orbitofrontal	MOrF	39, 40	Middle temporal	MT
9, 10	Pars opercularis	Op	41, 42	Superior temporal	ST
11, 12	Pars orbitalis	Or	43, 44	Temporal pole	TPol
13, 14	Pars triangularis	Tr	45, 46	Transverse temporal	TrT
15, 16	Rostral middle frontal	RoMF	**Parietal**		
17, 18	Superior frontal	SF	47, 48	Inferior parietal	IP
19, 20	Precentral gyrus	PreC	49, 50	Paracentral	PaC
**Limbic**			51, 52	Postcentral	PoC
21, 22	Caudal anterior cingulate	CACg	53, 54	Precuneus	PreCu
23, 24	Rostral anterior cingulate	RoACg	55, 56	Superior parietal	SP
25, 26	Isthmus cingulate	IstCg	57, 58	Supra marginal	SM
27, 28	Insula	Ins	**Occipital**		
29, 30	Parahippocampal	PaH	59, 60	Pericalcarine	PerCa
31, 32	Posterior cingulate	PoCg	61, 62	Fusiform	Fu
			63, 64	Cuneus	Cu
			65, 66	Lateral occipital	LO
			67, 68	Lingual	Lg

Figure S1: Regions in Desikan-Killiany Atlas. Retrieved from (Jao et al., 2021). Regions 1-34 correspond to left, and regions 35-68, correspond to right hemispheres.

REFERENCES

Gong, G., Rosa-Neto, P., Carbonell, F., Chen, Z. J., He, Y., and Evans, A. C. (2009). Age-and gender-related differences in the cortical anatomical network. *Journal of Neuroscience* 29, 15684–15693

Jao, C.-W., Lau, C. I., Lien, L.-M., Tsai, Y.-F., Chu, K.-E., Hsiao, C.-Y., et al. (2021). Using fractal dimension analysis with the desikan–killiany atlas to assess the effects of normal aging on subregional cortex alterations in adulthood. *Brain Sciences* 11, 107

Tzourio-Mazoyer, N., Landeau, B., Papathanassiou, D., Crivello, F., Etard, O., Delcroix, N., et al. (2002). Automated anatomical labeling of activations in spm using a macroscopic anatomical parcellation of the mni mri single-subject brain. *Neuroimage* 15, 273–289
